# Clinical associations for traditional and complementary medicine use among norwegian cancer survivors in the seventh survey of the Tromsø study: a cross-sectional study

**DOI:** 10.1186/s12906-023-03896-y

**Published:** 2023-03-04

**Authors:** Kiwumulo Nakandi, Trine Stub, Agnete E. Kristoffersen

**Affiliations:** National Research Center in Complementary and Alternative Medicine (NAFKAM), Faculty of Health Science, Department of Community Medicine, UiT The Arctic University of Norway, N-9037 Tromsø, Norway

**Keywords:** Cancer Survivor, Survivorship, Cancer Patient, Oncological patient, Traditional and complementary medicine, T&CM, Complementary and alternative medicine, CAM

## Abstract

**Background:**

Cancer survivors are a diverse group with varying needs that are patient-, disease-, and/or treatment-specific. Cancer survivors have reported supplementing conventional anti-cancer treatment with Traditional and Complementary Medicine (T&CM). Although female cancer survivors are reported to have more severe anticancer adverse effects, little is known about the association between anticancer treatment and T&CM use among Norwegian cancer survivors. The aims of this study are therefore to investigate (1) associations between cancer diagnosis characteristics and T&CM utilization and (2) associations between anticancer treatment and T&CM utilization among cancer survivors in the seventh survey of the Tromsø study.

**Methods:**

Data was collected from the seventh survey of the Tromsø Study conducted in 2015-16 among all inhabitants of Tromsø municipality aged 40 and above (response rate 65%), where inhabitants received online and paper form questionnaires. Data from the data linkage to the Cancer Registry of Norway for cancer diagnosis characteristics was also used. The final study sample was made up of 1307 participants with a cancer diagnosis. Categorical variables were compared using Pearson’s Chi-square test or Fisher’s exact test while independent sample t-test was used to compare continuous variables.

**Results:**

The use of T&CM the preceding 12 months was reported by 31.2% of the participants with natural remedies as the most reported modality of T&CM (18.2%, n = 238), followed by self-help practices of meditation, yoga, qigong, or tai chi, which was reported by 8.7% (n = 114). Users of T&CM were significantly younger (p = .001) and more likely to be female (p < .001) than the non-users, with higher use of T&CM among female survivors with poor self-reported health and being 1–5 years post-diagnosis. Lower use of T&CM was found among female survivors who received a combination of surgery with hormone therapy and those who received a combination of surgery with hormone therapy and radiotherapy. Similar usage was seen in male survivors, but not at a significant level. For both male and female survivors, T&CM was most frequently used by those with only one cancer diagnosis (p = .046).

**Conclusion:**

Our results indicate that the profile of the Norwegian cancer survivor who uses T&M is slightly changing compared to previous findings. Additionally, compared to male survivors, more clinical factors are associated with use of T&CM among female cancer survivors. These results should serve as a reminder to conventional health care providers to discuss the use of T&CM with patients across the entire cancer survivorship continuum to promote safe use, especially among female survivors.

## Background

In Norway, about 37 000 new cancer cases are recorded annually, with an overall growing number of cancer survivors. More males (53%) are diagnosed than females (47%), and the country’s median age at diagnosis is 70 years for both sexes [1]. Cancer survivorship is broadly defined as a continuum from the time of diagnosis to the end of one’s life [2]. Although this implies a commonality, cancer survivors are a diverse group with varying needs due to factors such as cancer type [3], stage at diagnosis [4], age at first diagnosis [5, 6], racial differences [7, 8] and disparities [9], and anti-cancer treatment [10, 11]. Cancer survivors also face adverse outcomes to varying degrees such as pain, fatigue, and gastrointestinal symptoms as a direct result of the disease [12]. Additionally, the most common conventional anticancer treatment forms such as surgery, chemotherapy, radiation, and hormonal therapy can have adverse effects [13–15]. The risk can increase when these modalities are used in combination [16].

Some effects, known as late effects of treatment, appear months to years after treatment [17]. These effects range from pain, fatigue, cardiotoxicity, lymphedema, neurological complications to secondary cancers [18, 19]. In addition to these physical effects, cancer survivors face psychological distress such as fear of recurrence which can result in anxiety, fear of death, and cognitive challenges [20]. To combat these effects, many cancer survivors report using Traditional and Complementary Medicine (T&CM) modalities [21–23].

T&CM can generally be defined as health practices not part of conventional medicine [24, 25]. Additionally, traditional medicine draws from the knowledge, beliefs, and century old experiences of indigenous peoples [26]. Other reasons given for the use of these modalities are to increase the body’s innate ability to fight the disease and to improve physical and mental well-being [27, 28]. This reflects the complex effects of cancer that survivors deal with, and possible insufficiencies in cancer survivorship care, more so if symptoms experienced are not recorded by health care providers as shown before [29].

The prevalence of T&CM utilization among cancer survivors varies from country to country from 16.5% in Italy to 93.4% in China [30–32]. The prevalence of T&CM utilization in Scandinavian countries like Sweden and Denmark ranges from 34 to 49% [28, 33]. In Norway where it is mainly used in addition to conventional anticancer treatment [34], prevalence of T&CM use among cancer survivors varies between 30% and 79% [35, 36]. Socio-demographic factors associated with T&CM use among European cancer survivors are female sex, higher education, and higher income [27, 37]. This is largely true for Norway too [36].

Use of T&CM before a cancer diagnosis has been shown to remain mainly unchanged upon diagnosis [28]. It can then decrease during treatment and return to pretreatment levels after anti-cancer treatment [38]. On the contrary, a remarkable increase in use upon diagnosis has also been shown [30]. Use among long-term survivors is also reported [39]. A breast cancer diagnosis [32], longer time since diagnosis [31, 40], advanced stage of disease [41], and metastasis [42] have also been positively associated with T&CM use. It was also shown that cancer survivors using T&CM were more likely to have a history of surgery and chemotherapy [43]. However, some studies have shown no correlation between clinical factors and T&CM use [38, 44].Greater severity of adverse events has been reported among female cancer survivors across different anticancer modalities [45], but little is known about how this affects the use of T&CM among Norwegian cancer survivors. The aims of this study are therefore to investigate (1) associations between cancer diagnosis characteristics and T&CM utilization and (2) associations between anticancer treatment and T&CM utilization among cancer survivors in the seventh survey of the Tromsø study.

## Methods

### Data collection

The data for this study were obtained from the seventh survey of the Tromsø study (Tromsø 7) and the Cancer Registry of Norway (CRN).

### The Tromsø study

**Fig. 1:**
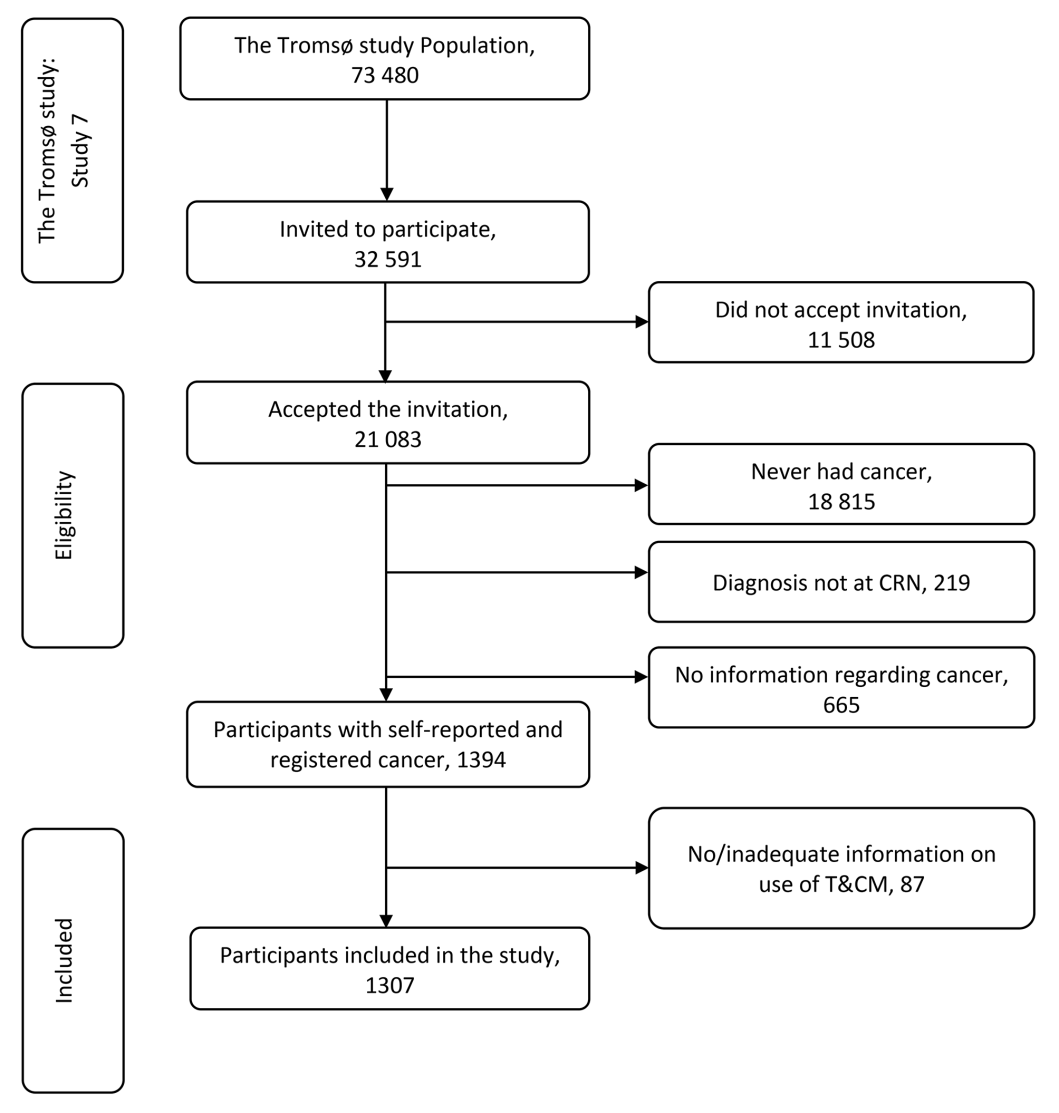
Flowchart of study participants

Tromsø, located in the Arctic, is the largest city in Northern Norway and is a center of education, research, and fishing [46]. The population is multicultural and is mainly made up of a Norwegian ethnic majority, Sami ethnic minority, and immigrants [47]. The Tromsø study is a prospective population-based study with repeated health surveys of the inhabitants in Tromsø, with seven surveys so far. Initially, the study was primarily about cardiovascular mortality, but now a multipurpose health survey [48]. The seventh survey of the Tromsø study (Tromsø7) has been described in detail elsewhere [49]. Briefly, for Tromsø7 (2015–2016), all inhabitants of the Tromsø municipality aged $\geqslant$40 years were invited (n = 32 591) to participate thorough a postal letter. The invitation included an information brochure and a personal letter with a username and password for completion of three online questionnaires (Q); a short general Q1; a long general Q2; and a graphical index of pain questionnaire. Q1 was also included as a 4-page paper questionnaire. The questionnaires, which were all in Norwegian with some in English, were a combination of different validated questionnaires and questions developed specifically for the Tromsø study. A total of 21 083individuals participated (64.7% of all invited, Fig. 1) [49].

### Cancer registry of Norway

The CRN, established in 1951, registers cancer cases, research on cancer, and activities related to cancer. Reporting to the CRN is mandatory for all medical doctors. Thus, the registry reports on cancer type, stage, other pathological characteristics, and treatment [50]. The registry has been found to have a high degree of comparability, accuracy, and timeliness [51].

Data harvested from the CRN used in this study comprised of cancer diagnosis (ICD-10 codes C00-D47), metastasis, date of diagnosis (multiple dates where relevant), and appurtenant treatments (several rounds of treatment where relevant).

### Data linkage

The Tromsø study links to national registries, including the CRN, through a unique 11-digit personal identification number assigned to all people residing in Norway.

### Study population

Our study population consisted of Tromsø7 participants with a self-reported cancer diagnosis that was registered at the CRN and adequate information on the use of T&CM. Firstly, 18 815 participants were excluded as they reported never having a cancer diagnosis (Fig. 1). A total of 219 participants reported a cancer diagnosis that was not registered with the CRN, so these participants were regarded as having no certain history of cancer and were excluded from the study. A further 655 were excluded due to missing information of self-reported history of cancer and were not registered with the CRN. The remaining 1394 participants all had a self-reported and a registered cancer diagnosis at the time of participation. Three participants with missing information on the use of T&CM were also excluded. To avoid bias, a total of 84 participants who answered “no” to the use of one type of T&CM and had missing information on the remaining modalities of T&CM were excluded as the use/non-use of T&CM could not be established. The final study sample was made up of 1307 participants who had a self-reported cancer diagnosis that was registered at the CRN and provided adequate information on the use of T&CM.

### Basic characteristics

The four-page paper questionnaire (Q1) sent along with the invitation letter and an additional online survey (Q2) were used to collect the demographic (age, sex, living with a spouse/partner, education, and household income) and health information of the participants.

### Traditional and complementary medicine use

Users of T&CM were defined as participants who reported visits to a T&CM provider and/or used non-provider interventions in the preceding 12 months. Simplified questions based on the international questionnaire to measure use of complementary and alternative medicine questions, the I-CAM-Q [52] were used. The use of a T&CM provider was based on a “yes” response to either of these three questions from Q1: “Have you during the past year visited a traditional healer (helper, “reader”, etc.?)”, “Have you during the past year visited an acupuncturist?” or “Have you during the past year visited a complementary medicine provider (homeopath, reflexologist, spiritual healer, etc.?)”. Non-provider T&CM use was collected from Q2 through “Have you used herbal medicines, natural remedies, or herbal remedies during the last 12 months?” (natural remedies henceforth), and “Have you used meditation, yoga, qigong, or tai chi as self-treatment during the last 12 months?” (self-help practices henceforth) with the response options “yes” and “no”. Data on T&CM use was self-reported.

### Health information

Data about self-reported cancer was obtained from Q1 through the question: “Have you ever had, or do you have cancer?” with the alternatives “no”, “yes, now” and “previously, not now”.

Self-reported health was collected through a categorical variable with five categories, sourced from Q1 that asked: “How do you in general consider your own health to be?” with the following answer alternatives; “very bad”, “bad”, “neither good nor bad”, “good”, and “excellent”. These were compressed to, “bad” (very bad and bad), “neither good nor bad”, and “good” (good and excellent).

### Cancer diagnosis characteristics

Diagnosis per ICD-10 from the CRN was regrouped into cancer site of the body to create the variables, “Breast”, “Female genital organs”, “Prostate”, “Other male genital organs”, “Lung and other respiratory and intrathoracic organs”, “Melanoma and skin”, “Colon”, “Other digestive organs”, “Lymphoid”, “Hematopoietic and related tissue”, and “Other cancers”.

“Time since first diagnosis” was understood as the period since the first diagnosis date and the date of participation. It was used as a continuous variable to calculate the mean number of years since the first diagnosis and further merged into a categorical variable and grouped into 4 periods, “less than a year”, “1–5 years”, “6–10 years” and “more than 10 years”.

Metastasis was divided into 3 groups: metastasis, no metastasis, and unknown.

### Type of treatment

Types of treatment were obtained from the CRN and re-grouped as follows:

Surgical treatment was understood as a surgical procedure made with the intent of treating the disease [53]. As such, registered diagnostic procedures (exploratory and biopsies) were not included. Surgical treatment for the first up to a fourth diagnosis was combined to create the variable for “Surgery”.

Chemotherapy meant any kind of registered chemotherapeutical treatment [54]. Chemotherapy for the first up to a fourth diagnosis was combined to create the variable for “Chemotherapy”.

Radiation therapy was understood as all forms of radiation, including the gamma knife and radioactive iodine [55]. Radiation treatment for the first up to a fourth diagnosis was combined to create the variable for “Radiotherapy”.

Hormonal therapy meant having received any kind of hormonal therapy, with or without the combination of other therapies/procedures [56]. Hormonal therapy for the first up to a fourth diagnosis was combined to create the variable for “Hormone therapy”.

Multimodal treatments were understood as receiving more than one form of the treatments above (regardless of times one has been diagnosed with cancer). This led to the following combinations, “Surgery and chemotherapy”, “Surgery and radiation”, “Surgery and hormonal therapy”, “Chemotherapy and radiation”, “Chemotherapy and hormonal therapy”, “Surgery, chemotherapy and hormonal therapy”, “Chemotherapy, hormonal therapy and radiation” “Surgery, chemotherapy and radiation”, “Surgery, chemotherapy, radiation and hormonal therapy”.

### Statistical analysis

Descriptive statistics were carried out using cross-tabulation and frequency analyses. Categorical variables were compared using Pearson’s Chi-square test or Fisher’s exact test while independent sample t-test was used to compare continuous variables. With a margin of error of 5%, a confidence level of 95%, and a heterogeneity of 50%, we needed a minimum sample of n = 384 to represent the Norwegian cancer population of 262 884 for adequate study power. Statistical significance was set at the p-value of < 0.05. All statistical analyses were done using Statistical Package for Social Sciences (SPSS) version 28.0 for Windows.

## Results

### Basic characteristics

The study population consisted of 1307 cancer survivors, 655 females (50.1%) and 652 males (49.9%). The average age of the participants was 65.6 years (SD= 10.7). A total of 74.4% of the participants reported living with a spouse. Most of the participants reported attaining tertiary education (46.8%) and reported medium and high income (81.7%, Table 1).

**Table 1:** Basic Characteristics of the participants and association for T&CM use

	Total, n = 1307	%	T&CM, n = 408	%	No T&CM, n = 899	%	p-value
**Sex**							**< 0.001**
Females	655	50.1	242	59.3	413	45.9	
Males	652	49.9	166	40.7	486	54.1	
**Age**							**0.001**
40–62	456	34.9	168	41.2	288	32.0	
63 and above	851	65.1	240	58.8	611	68.0	
Mean age (SD)	65.61 (10.742)		63.97 (11.325)		66.36 (10.388)		**0.011**
**Education**							0.406
Primary level	382	29.2	110	27.0	272	30.3	
Secondary level	313	23.9	97	23.8	216	24.0	
Tertiary	612	46.8	201	49.3	411	45.7	
**Income**							0.824
Low	228	18.3	72	18.7	156	18.1	
Medium	567	45.5	170	44.2	397	46.1	
High	452	36.2	143	37.1	309	35.8	
**Living with a spouse/partner**							
Yes	931	74.4	288	74.0	643	74.5	0.860
No	321	25.6	101	26.0	220	25.5	

### Disease characteristics

The most common cancers were prostate cancer (21.9%) and breast cancer (20.0%), followed by melanoma and other skin cancers (9.7%, Table 2). Metastatic disease was found in 30.6% of the participants and 0.2% had an unknown metastatic status at diagnosis. The mean time since the first cancer diagnosis was 9.2 years (SD= 9.29). Most participants had one cancer diagnosis only, n = 1067 (81.6%), and 240 people had 2 or more consecutive cancer diagnoses (18.4%, Table 2).

**Table 2:** Clinical characteristics and associations for T&CM use

	Total N = 1307	%	T&CM, N = 408	%	No T&CM, N = 899	%	p-value
**Self-reported health**							0.283
Medium to good	1207	92.3	372	91.2	835	92.9	
Poor	100	7.7	36	8.8	64	7.1	
**Cancer site first diagnosis**							0.309
Breast	262	20.0	98	24.0	164	18.2	
Female genital organs	105	8.0	32	7.8	73	8.1	
Prostate	286	21.9	74	18.1	212	23.6	
Other male genital organs	49	3.7	14	3.4	35	3.9	
Respiratory organs	31	2.4	9	2.2	22	2.4	
Melanoma and skin	127	9.7	42	10.3	85	9.5	
Colon	99	7.6	29	7.1	70	7.8	
Other digestive organs	72	5.5	23	5.6	49	5.5	
Hematologic*	93	7.1	25	6.1	68	7.6	
Other cancers	183	14.0	62	15.1	121	13.5	
**Metastasis at first diagnosis** ^**!**^							0.779
Metastasis	334	30.6	109	31.5	225	30.2	
No metastasis	754	69.2	236	68.2	518	69.6	
Unknown	2	0.2	1	0.3	1	0.1	
**Time since first cancer diagnosis**							0.072
less than a year	117	9.0	38	9.3	79	8.8	
1–5 years	485	37.1	167	40.9	318	35.4	
6–10 years	265	20.3	86	21.1	179	19.9	
more than 10 years	440	33.7	117	28.7	323	35.9	
Mean time since first cancer diagnosis (SD)	9.20 (9.299)		8.57 (9.489)		9.49 (9.202)		0.098
**Age at first cancer diagnosis**							0.132
0–20 years	31	2.7	7	1.9	24	3.1	
21–49 years	344	30.0	122	33.6	222	28.4	
50 years and above	770	67.2	234	64.5	536	68.5	
**Number of cancer diagnoses**							.**046**
Cancer once	1067	81.6	346	84.8	721	80.2	
Cancer twice or more times	240	18.4	62	15.2	178	19.8	
**Only one treatment modality** ^**!**^							0.340
Surgery	184	41.2	54	36.0	130	43.8	
Chemotherapy	57	12.8	19	12.7	38	12.8	
Radiotherapy	186	41.6	71	47.3	115	38.7	
Hormone therapy	20	4.5	6	4.0	14	4.7	
**Multimodal treatment** ^**!**^							
Surgery and CT	372	28.5	107	26.2	265	29.5	0.227
Surgery and RT	523	40.0	165	40.4	358	39.8	0.832
Surgery and HT	274	21.0	70	17.2	204	22.7	0.023
CT and RT	231	17.7	67	16.4	164	18.2	0.424
CT and HT	193	14.8	58	14.2	135	15.0	0.705
Surgery, CT and HT	322	24.6	93	22.8	229	25.5	0.298
CT, HT and RT	236	18.1	69	16.9	167	18.6	0.469
Surgery, HT and RT	293	22.4	75	18.4	218	24.2	0.018
Surgery, CT and RT	336	25.7	95	23.3	241	26.8	0.177
Surgery, CT, RT and HT	337	25.8	96	23.5	241	26.8	0.209

*Lymphoid, hematopoietic, and related tissue; ^**!**^n is not equal to 1307 due to missing data; CT: Chemotherapy; RT: Radiotherapy; HT: Hormone therapy

### Prevalence of T&CM

Overall use of T&CM was reported by 31.2%. Users of T&CM were significantly younger than non-users, 64.0 (SD= 11.33) and 66.4 (SD= 10.39) years respectively (p = .011, Table 1). Natural remedies were the most reported modality of T&CM and were reported by 18.2% (n = 238) of the participants, followed by self-help practices which was reported by 8.7% (n = 114). Provider-based T&CM modalities were used by 10.0% (n = 131) and were each (acupuncturists; traditional healers; and other complementary medicine providers) reported by 4.0% of the participants (Table 3). T&CM users were more likely to be female survivors, making up almost 60% (p < .001, Table 1). Female T&CM users were also more likely to visit a T&CM provider compared to male T&CM users, but not at a significant level (11.6% vs. 8.4%, p = .057). However, they reported more use of self-help practices compared to male survivors (14.1% vs. 3.4 % respectively, p < .001, Table 3).

**Table 3:** Use of different types of T&CM

Type of T&CM	Number of users	%	Female, n = 655	%	Male, n = 652	%	p-value
Acupuncturist	54	4.2	36	5.6	18	2.8	0.012
Traditional Healer	52	4.0	22	3.5	30	4.7	0.282
Other CM providers	52	4.0	27	4.2	25	3.9	0.760
Provider-based*	131	10.0	76	11.6	55	8.4	0.057
Natural remedies	238	18.3	130	19.9	108	16.6	0.118
Self-practices**	114	8.7	92	14.1	22	3.4	< 0.001

*Provider-based: Total users of Acupuncturist, other Complementary Medicine Provider, and or Traditional Healer;**Self-practices = Meditation, Yoga, Qi Jong or Tai Chi. T&CM: Traditional and Complementary Medicine

**Table 4:** Clinical associations of T&CM use

	Female, n = 655					Male, n = 652				
	T&CM, n = 242	%	No T&CM, n = 413	%	p-value	T&CM n = 166	%	No T&CM n = 486	%	p-value
**Self-reported health**					0.038					0.341
Medium to good	217	88.8	386	93.5		157	94.6	449	92.4	
Poor	27	11.2	27	6.5		9	5.4	37	7.6	
**Cancer site first diagnosis**					0.876					0.954
Breast	98	40.5	164	39.7		-		-		
Female genital organs	32	13.2	73	17.7		-		-		
Prostate	-		-			74	44.6	212	43.6	
Other male genital organs	-		-			14	8.4	35	7.2	
Respiratory organs	7	2.9	11	2.7		2	1.2	11	2.3	
Melanoma and skin	24	9.9	36	8.7		18	10.8	49	10.1	
Colon	16	6.6	31	7.5		13	7.8	39	8.0	
Other digestive organs	12	5.0	19	4.6		11	6.6	30	6.2	
Hematologic*	14	5.8	23	5.6		11	6.6	45	9.3	
Other cancers	39	16.1	56	13.6		23	13.9	65	13.4	
**Breast cancer**					0.843					
Breast	98	40.5	144	39.7						
Other sites	144	59.5	249	60.3						
**Prostate cancer**										0.830
Prostate						74	44.6	212	43.6	
Other sites						92	55.4	274	56.1	
**Metastasis at first diagnosis**					0.254					0.680
Metastasis	71	34.1	104	30.1		38	27.5	121	30.4	
No metastasis	136	65.4	242	69.9		100	72.5	276	69.3	
Unknown	1	0.5	0	0.0		0	0.0	1	0.3	
**Time since first cancer diagnosis**					**0.002**					0.703
less than a year	21	8.7	29	7.0		17	10.2	50	10.3	
1–5 years	100	41.3	118	28.6		67	40.4	200	41.2	
6–10 years	48	19.8	87	21.1		38	22.9	92	18.9	
more than 10 years	73	30.2	179	43.3		44	26.5	144	29.6	
**Number of cancer diagnoses**					0.132					0.303
Cancer once	209	86.4	338	81.8		137	82.5	383	78.8	
Cancer twice or more times	33	13.6	75	18.2		29	17.5	103	21.2	
**Only one treatment modality**					0.372					.559^1^
Surgery	28	27.2	45	31.3		26	55.3	85	55.6	
CT	12	11.7	18	12.5		7	14.9	20	13.1	
RT	60	58.3	71	49.3		11	23.4	44	28.8	
HT	3	2.9	10	6.9		3	6.4	4	2.6	
**Multimodal treatment**										
Surgery and CT	58	24.0	121	29.3	0.140	49	29.5	144	29.6	0.978
Surgery and RT	112	46.3	189	45.8	0.898	53	31.9	169	34.8	0.503
Surgery and HT	38	15.7	101	24.5	**0.008**	32	19.3	103	21.1	0.599
CT and RT	51	21.1	93	22.5	0.667	16	9.6	71	14.6	0.104
CT and HT	43	17.8	75	18.2	0.900	15	9.0	60	12.3	0.249
Surgery, CT and HT	59	24.4	113	27.4	0.403	34	20.5	116	23.9	0.371
CT, HT and RT	52	21.5	94	22.8	0.706	17	10.2	73	15.0	0.123
Surgery, HT and RT	41	16.9	106	25.7	**0.010**	34	20.5	112	23.0	0.494
Surgery, CT and RT	61	25.2	119	28.8	0.318	34	20.5	122	251	0.228
Surgery, CT, RT and HT	61	25.2	119	28.8	0.318	35	21.1	122	25.1	0.296

T&CM: Traditional and Complementary Medicine. *Hematologic = Lymphoid, hematopoietic, and related tissue. ^1^Fisher-Freeman-Halton Exact Test. CT: Chemotherapy; RT: Radiotherapy; HT: Hormone therapy

### Associations between cancer diagnosis characteristics and T&CM use

#### Female cancer survivors

Female cancer survivors with poor self-reported health were more likely to use T&CM (11.2%) compared to non-users of T&CM with poor self-reported health (6.5%, p = .038). Although most female T&CM users were breast cancer survivors (40.5%), there were no significant differences in cancer site and T&CM utilization among female participants (p = .876, Table 4). This remained when type of cancer was analyzed as a binary variable (“breast cancer” and “other sites”, p = .843, Table 4). There were no significant differences in metastatic disease or number of cancer diagnoses and T&CM use among female cancer survivors. Most of the female T&CM users (41.3%) were 1–5 years post-diagnosis, compared to 8.7% who were 19.8% who were 6–10 years post-diagnosis, and 30.2% who were more than 10 years post-diagnosis (p = .002).

#### Male cancer survivors

T&CM use was similarly distributed across cancer sites among male cancer survivors (p = .945); however, ranging from 19.6% (hematologic cancer) to 26.8% (male genital organs other than prostate). We found no significant associations in metastatic disease, time since diagnosis and number of diagnoses among male cancer survivors.

### Associations between anticancer treatment and T&CM use

Receiving a combination of surgery and hormone therapy was negatively associated with the use of T&CM among female cancer survivors (15.7% of T&CM users vs. 24.5% of non-T&CM users, p = .025). A combination of surgery, hormone, and radiotherapy was also negatively associated with the use of T&CM among female cancer survivors (16.9% among T&CM users vs. 25.7% among non-T&CM users, p = .010). There were otherwise no significant associations found between type of anticancer treatment (multimodal or individual) and T&CM use among female cancer survivors. Among male cancer survivors, we found no associations between receiving one or multimodal anticancer treatment and the use of T&CM (Table 4).

## Discussion

This study revealed that 31.2% of the participants reported having used T&CM in the preceding year with higher use among female cancer survivors 1–5 years post-diagnosis and with poor self-reported health. Surgery and hormone therapy, as well as surgery, hormone therapy and radiotherapy in combination were negatively associated with the use of T&CM among female survivors.

### Changes in cancer diagnosis characteristics and T&CM use from the Tromsø6 to the Tromsø7 study

The findings in this study differ somewhat from the findings of a Tromsø6 study conducted 6 years earlier (2012) in the same population [57]. The current study showed no statistically significant association between the use of T&CM and type of cancer, neither among female nor male survivors. This contrasts with the Tromsø6 study where breast cancer survivors used more T&CM than female survivors with other cancer forms. The reason for this difference could be due to a decrease in T&CM use among breast cancer survivors, leveling out the prevalence of T&CM use among female survivors of breast cancer and other cancer sites. The reason for this decrease could be improvement in breast cancer detection and more targeted anticancer treatments [58], as this may reduce the adverse effects and late effects of the cancer diagnosis and treatment, leading to less need for T&CM. This is suspected as treating adverse effects and late and long term effects of cancer diagnosis and treatment is one of the main reasons why Norwegian cancer patients use T&CM [36]. Furthermore, in contrast to no associations between the use of T&CM and time since diagnosis in Tromsø6, findings in the current study revealed more use of T&CM among short term survivors than acute and long term survivors [57]. This difference could be due to the smaller sample size in Tromsø6 leading to less power in the statistical analyses as similar tendencies are seen in Tromsø6 and7, even where no statistical significance is achieved. There were no associations found between the use of T&CM and metastasis, neither in Tromsø6 nor Tromsø7.  . This is in contrast to other findings across Europe [27]. This could be due to the high number of medium – good self-reported health among the participants of the current study that might have removed the need for T&CM even among survivors with metastatic cancer. 

### Other comparable studies

Incidence of types of cancer differs globally, as does cancer mortality [59]. Additionally, availability and access to conventional cancer treatments also vary [60]. T&CM modalities are primarily used as a supplement to conventional treatment in the Western hemisphere, but are used as primary treatment in some areas around the globe [60]. Thus, meaningful international cross-study comparison of clinical associations of T&CM is hard to achieve. For valid comparability, we compare our study to studies carried out in regions with a similar health service landscape, as well as similar access to it. Universal health care exists in all Europe, with the Nordic model (publicly financed comprehensive health care systems) being used in Norway [61].

We found differences in the clinical characteristics associated with T&CM among female and male survivors. This could be due to sex differences in the psychological and physical effects of the disease [62], and differences in treatment strategies for various reasons like gender bias (where no apparent medical reason justifies why female patients are not offered the same treatment as males [63]). For example, a recent study showed that female rectal cancer survivors were less likely to receive preoperative radiotherapy than male survivors of the same age, level of comorbidity, and tumor depth [64]. Furthermore, female survivors have been associated with greater cancer-related distress [65]. Additionally, some diffuse and chronic disorders commonly associated with women as a group still get less attention within the healthcare system [62]. These factors could lead female survivors to seek T&CM to a higher degree than male survivors to compensate for the differences in the treatment options not offered, or health burdens not adequately addressed. Sex or gender differences in utilization of T&CM have otherwise been discussed as a “gender puzzle” that might be a result of social constructs and healthcare inequalities. As women are more familiar with physical and emotional care and dispense it more than men, they engage and receive T&CM approaches easier compared to men.. Women were also reported to use alternative health services to avoid power-imbalances in conventional health services [66].

It comes as no surprise that poor self-reported health was associated with T&CM use as the perception of health and illness are major motivations for health care utilization [67]. Poor self-reported health has been associated with use of T&CM [68, 69] and with higher utilization of health services in general [70], more so among women than men [71]. Poor health possibly opens survivors up to supplementing conventional treatment to improve their health. Indeed, a recent Norwegian study exploring the rationale for T&CM use among cancer survivors found that most survivors used T&CM to increase quality of life and well-being [36].

Female participants 1–5 years post the first cancer diagnosis (short-term survivors) were more likely to use T&CM than those less than a year (acute survivors), and more than 5 years post-diagnosis (long-term survivors). This is in accordance with a study from the U.S. that found more use of T&CM self-help practices among short-term survivors compared to acute and long-term survivors [68]. Our findings of higher T&CM use among short-term survivors are, on the other hand, both similar and discordant to earlier Norwegian studies. The reason for the higher use among short-term survivors in the current study might be multifaceted. One reason might be the fact that the majority of cancer survivors report unmet physical, emotional and practical concerns post anti-cancer treatment [72], opening them up to the use of non-conventional health services. Additionally, they report a sense of abandonment after discharge [73], a loss of a safety net, and decline in interpersonal support in this phase [74]. These factors might have driven the short-term cancer survivors to use T&CM to a larger degree than acute survivors who are followed by a multidisciplinary team at varying frequencies [75]. Long-term survivors could, on the other hand, have adapted better to their diagnoses with time [76]. Although the Norwegian cancer patient pathway entails follow-up such as rehabilitation [77] that would help attend to survivors’ concerns in this phase, a study showed that the post-treatment/rehabilitation period is assigned low priority by professionals due to acute or urgent tasks like palliative care [78]. Additionally, cancer survivorship care is not yet formalized and part of conventional health professional schools’ curricula [79]. Breast cancer survivorship care in Norway, for example, is organized by the hospital that treated the survivor for the first two years with a recommended doctor’s consultation. The third and fourth year follow-up entails telephone consultations or nurse consultations at the hospital that treated the survivor, as well as a clinical examination with a primary or tertiary physician [80]. This can be perceived as inadequate healthcare by some, more so by survivors who use T&CM as they have been associated with high-use behavior of conventional health care services [34]. This mismatch of growing health concerns and a less active role of conventional health care providers might make T&CM especially attractive during this transition to life after active anticancer treatment. Beyond unaddressed concerns from health care providers, the short-term survivorship period poses new challenges for the survivor. Although anticancer treatment might be completed and successful, survivors enter an unfamiliar phase with fear of recurrence, anxiety, and treatment-related or new symptoms [74]. T&CM has therefore been used in this phase to promote health and well-being, prolong life after active treatment, and for disease prevention [73, 81].

In this study, we found negative as well as no significant associations between the use of T&CM and conventional anti-cancer treatment. This contrasts with previous studies that found T&CM more commonly used among survivors with a history of chemotherapy [43, 76] and surgery [43]. The reason for this difference in findings could be twofold. The informants in one of the studies were undergoing conventional anti-cancer treatment at the time of the survey or had received it no more than three years prior to the survey. The participants in our study were mostly long-term survivors, and therefore, the majority were unlikely to be undergoing anti-cancer treatment at the time of participation. Additionally, we investigated T&CM use in the preceding 12 months, mostly long after the anti-cancer treatment took place. Berretta et al. also reported higher use of T&CM (48.9%) [76], which is higher than that found in our study and might have led to different associations for use.

### Strengths and limitations

One of the major strengths of this study is the large study population and rather high response rate and that self-reported cancer was confirmed by a diagnosis recorded at the CRN. This eliminated recall bias in terms of the time of diagnosis, type of cancer, metastatic disease, and treatments used. The cohort was also well-balanced with 1:1 ratio of female and male participants. As the questionnaire was linked to a wider population-based health survey, there was minimal risk of self-selection bias to disproportionately attract T&CM users to participate. The sample in the present study also reflected cancer site incidence in Norway at the time of the study [82]. Additionally, as the study took place outside a hospital/treatment center setting, the cohort included survivors at different phases of their diagnosis and treatment.

The CRN has an extensive overview of patients’ therapies, but due to low completeness of administered cancer therapies [83], data on several of the treatment modalities was lacking. Thus, interpretation of treatment-related associations of T&CM use should be with caution.

Even though the questionnaire captures the different forms of T&CM modalities like provider-based, natural remedies, and self-help practices, the questions go ahead to specify the different types, but the lists were not exhaustive. This might have led to confusion on how to understand T&CM and led to underreported use of T&CM modalities not listed. The modalities listed could also have led to misunderstanding and consequently incorrect reporting of their use.

It is unlikely that terminally ill cancer survivors participated in this study as the study took place outside a hospital/treatment center setting, and few patients reported very poor health. Additionally, some data on metastasis was missing. Thus, the association between disease severity and the use of T&CM, or lack thereof, could be non-representative for the total population.

Although the cross-sectional design highlights associations of T&CM use, it limits inferences of causality of T&CM use among the participants. Indeed, knowledge on reasons for T&CM use would improve the interpretation of the clinical associations of T&CM use among cancer survivors. The reader should note that few of the participants of this study were acute cancer survivors, so the generalizability of these findings applies more to short and long-term cancer survivors. Finally, even though the self-reported use of T&CM regarded use in the preceding 12 months, recall bias could still influence the participants’ answers. We argue that there would be a realistic reporting of provider-based T&CM as these modalities entail meeting the provider and could be easier to recall, but a possible underreporting of non-provider based/self-management T&CM. On the other hand, the study taking place outside a hospital/treatment center setting could have removed any fear to report any use of T&CM and allowed for more honest answers.

### Implications of the study

Our findings add to the existing literature on the use of T&CM among Norwegian cancer survivors and have clinical and research implications. Conventional health care providers should be more sensitive to these clinical associations of T&CM in interactions with survivors and initiate bias-free conversations about T&CM use. More so as cancer survivors appreciate open communication about T&CM with their conventional health care providers [84]. This can also help ward off the use of modalities without proven safety. Further research could examine why there is an increase in T&CM use 1–5 years post-diagnosis among female cancer survivors. This would help address unmet concerns among female survivors and add to the growing knowledge on sex differences in health care utilization. Further, this could also help guide policymakers in the continued development of health equality of survivorship care programs, as well as medical education programs for professionals. As the participants of this study are 40 years and above, similar studies are needed among younger cancer survivors. Understanding clinical factors associated with T&CM use among cancer survivors of all ages can uncover weaknesses in the conventional health care system while strengthening survivorship care.

## Conclusion

The findings indicate that the profile of the Norwegian cancer survivor who uses T&M is slightly changing compared to findings six years earlier. Additionally, compared to male survivors, more clinical factors are associated with the use of T&CM among female cancer survivors. These results should serve as a reminder to conventional health care providers to discuss the use of T&CM with patients across the entire cancer survivorship continuum to promote safe use, especially among female survivors.

## Data Availability

The raw dataset is not available due to Norwegian privacy regulations. Applicants for any data must be prepared to conform to Norwegian privacy regulations.

## Abbreviations

T&CM: Traditional and Complementary Medicine
SD: Standard Deviation
CRN: Cancer Registry of Norway REK:Regional Committee of Medical and Health Research Ethics
UiT: University of Tromsø.
